# Abnormal promoter DNA hypermethylation of the integrin, nidogen, and dystroglycan genes in breast cancer

**DOI:** 10.1038/s41598-021-81851-y

**Published:** 2021-01-26

**Authors:** Vladimir V. Strelnikov, Ekaterina B. Kuznetsova, Alexander S. Tanas, Viktoria V. Rudenko, Alexey I. Kalinkin, Elena V. Poddubskaya, Tatiana V. Kekeeva, Galina G. Chesnokova, Ivan D. Trotsenko, Sergey S. Larin, Sergey I. Kutsev, Dmitry V. Zaletaev, Marina V. Nemtsova, Olga A. Simonova

**Affiliations:** 1 Epigenetics Laboratory, Research Centre for Medical Genetics, Moskvorechie St 1, 115522 Moscow, Russia; 2 Molecular Genetic Diagnostics Laboratory 2, Research Centre for Medical Genetics, Moskvorechie St 1, 115522 Moscow, Russia; 3 Medical Genetics Laboratory, I.M. Sechenov First Moscow State Medical University (Sechenov University), Trubetskaya St 8-2, 119991 Moscow, Russia; 4 Clinic of Personalized Medicine, I.M. Sechenov First Moscow State Medical University (Sechenov University), Trubetskaya St 8-2, 119991 Moscow, Russia; 5 VitaMed LLC, Seslavinskaya St 10, 121309 Moscow, Russia; 6 Institute of Medicine, Peoples’ Friendship University of Russia (RUDN University), Miklukho-Maklaya St 6, 117198 Moscow, Russia; 7 Molecular Immunology Laboratory, Federal Scientific Clinical Centre of Pediatric Hematology Oncology Immunology Named After Dmitry Rogachev, Samory Mashela St 1, 117997 Moscow, Russia; 8 Gene Therapy Laboratory, Institute of Gene Biology, Vavilova St 34/5, 119334 Moscow, Russia

**Keywords:** Cancer, Genetics, Biomarkers, Molecular medicine, Oncology

## Abstract

Cell transmembrane receptors and extracellular matrix components play a pivotal role in regulating cell activity and providing for the concerted integration of cells in the tissue structures. We have assessed DNA methylation in the promoter regions of eight integrin genes, two nidogen genes, and the dystroglycan gene in normal breast tissues and breast carcinomas (BC). The protein products of these genes interact with the basement membrane proteins LAMA1, LAMA2, and LAMB1; abnormal hypermethylation of the *LAMA1*, *LAMA2*, and *LAMB1* promoters in BC has been described in our previous publications. In the present study, the frequencies of abnormal promoter hypermethylation in BC were 13% for *ITGA1*, 31% for *ITGA4*, 4% for *ITGA7*, 39% for *ITGA9*, 38% for *NID1*, and 41% for *NID2*. *ITGA2, ITGA3, ITGA6, ITGB1*, and *DAG1* promoters were nonmethylated in normal and BC samples. *ITGA4, ITGA9*, and *NID1* promoter hypermethylation was associated with the HER2 positive tumors, and promoter hypermethylation of *ITGA1, ITGA9*, *NID1* and *NID2* was associated with a genome-wide CpG island hypermethylated BC subtype. Given that *ITGA4* is not expressed in normal breast, one might suggest that its abnormal promoter hypermethylation in cancer is non-functional and is thus merely a passenger epimutation. Yet, this assumption is not supported by our finding that it is not associated with a hypermethylated BC subtype. *ITGA4* acquires expression in a subset of breast carcinomas, and methylation of its promoter may be preventive against expression in some tumors. Strong association of abnormal *ITGA4* hypermethylation with the HER2 positive tumors (p = 0.0025) suggests that simultaneous presence of both HER2 and integrin α4 receptors is not beneficial for tumor cells. This may imply HER2 and integrin α4 signaling pathways interactions that are yet to be discovered.

## Introduction

Cell transmembrane receptors and extracellular matrix components play a pivotal role in regulating cell functioning and ensure concerted cell integration in the tissue structure. Malignant transformation is associated with dramatic alterations in cell signaling. Such alterations affect both cancer cell behavior and components of the tumor microenvironment. In turn, microenvironmental components may both promote and suppress the pathological process^1^.

**Figure 1:**
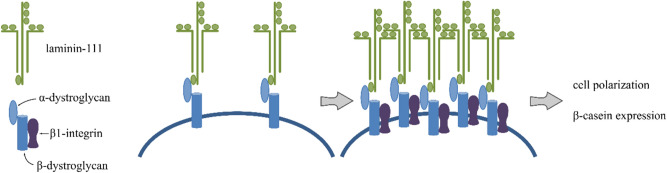
Laminins, integrins, and dystroglycan interactions are considered prerequisite for polarization and normal functioning of the mammary cell.

Concerted integration of morphogenetic signals and proper cell adhesion and migration are essential for preserving and maintaining the tissue structure by cells. Regulatory mechanisms include ligand–receptor interactions of cells with surrounding components. The interactions that involve laminins, integrins, and dystroglycan (Fig. 1) are critical for the function of breast epithelial cells^2^.

The integrin family is one of the important classes of cell transmembrane receptors. Structurally, integrins are heterodimers of α and β subunits, which are noncovalently bound with each other. A total of 18 α and 8 β subunits are known today. Their pairwise interactions produce at least 24 αβ receptor combinations. As their important functions, integrins mediate bidirectional signaling and contribute to cell adhesion by forming hemidesmosomes and focal contacts. Integrins trigger various regulatory cascades by converting external signals to internal ones. Integrins are capable of affecting various cell processes, such as growth, differentiation, adhesion, migration, and apoptosis^3^.

Given that multiple functions are mediated by integrins, one can assume that integrins are involved in cell malignant transformation, invasion, and metastasis. Ample data have accumulated to date to characterize the role that integrins play in morphogenesis and function of the breast epithelium, and changes in integrin expression have been observed in BC^4^.

We have previously examined three laminin subunit genes (*LAMA1, LAMA2*, and *LAMB1*), the promoter regions of which undergo abnormal methylation in breast cancer (BC) at frequencies higher than 16%^5^. In the present study, we drew attention to the genes encoding proteins that can interact with laminin molecules that incorporate the products of the *LAMA1*, *LAMA2*, *LAMB1* genes. The group of interacting molecules includes integrin subunits *ITGA1*, *ITGA2*, *ITGA3*, *ITGA6*, *ITGA7*, *ITGA9*, *ITGB1* genes, a dystroglycan (*DAG1*) gene^6,7^, and nidogens *NID1* and *NID2* genes^8,9^. *ITGA4,* also included in this study, settles somewhat aside from these genes: in some publications, the possibility of interaction of its product with laminin-111 was described; however, it is currently believed that interaction with laminins is not its main function^10^. Yet, data has accumulated indicating its involvement in a variety of cancers including BC^11–14^, which was the reason for its inclusion.

To date, most detailed information in terms of tumorigenesis is available for two integrin subunits selected for this study, ITGA9 and ITGA4. ITGA9 is a component of integrin α9β1, which is capable of interacting with many molecules, including thrombospondin 1, ADAM12/ADAM15, the nerve growth factor, VCAM1, laminin, fibronectin, tenascin C, osteopontin, VEGF-C, and VEGF-D^15^. *ITGA9* expression is vital, and *ITGA9* knockout mice die on day 6–12 of embryo development. Integrin α9β1 is involved in regulating cell adhesion, migration, differentiation, and proliferation. The role that integrin α9 plays in carcinogenesis has been the focus of many studies. Upregulation of integrin α9 expression has been observed in medulloblastoma^16^, astrocytoma, and glioblastoma^17^. On the other hand, a NotI microarray study of genetic and epigenetic *ITGA9* alterations has shown that its deletions and methylation occur at a high (> 30%) frequency in renal cell, lung, breast, ovarian, cervical, and rectal cancers. In breast tumors, *ITGA9* expression was downregulated or totally absent in 44% of cases and intact or upregulated in 45% of cases. The frequency of *ITGA9* promoter methylation was 90% in the former group. Treatment with the demethylating agent 5-aza-dC restored integrin α9 expression in ITGA9-negative cells of the MCF7 line^18^. A study of cervical carcinoma has detected *ITGA9* deletions in 41% of cases and abnormal promoter methylation in 24% of cases^19^. It is important to note that *ITGA9* is in the so-called AP20 region of chromosome 3p21.3. The region is a hot spot of homozygous deletions in renal cell, lung, and breast carcinomas and is involved in malignant transformation of various epithelial cells^20^. Apart from deletions, extended fragments with an altered methylation status and modified chromatin have been observed in this region in rectal tumors^21^. Interesting observations have been made in a large pedigree with Lynch syndrome. Its members suffered from various cancers, including endometrial, rectal, hepatic, gastric, breast, and brain cancers; leukemia; etc. A molecular testing revealed a large 400-kb deletion involving *LRRFIP2, GOLGA4*, and *C3orf35/APRG1*. The deletion produced a fusion gene combining *MLH1* exons 1–11 with *ITGA9* exons 17–28. It is thought that the 3′-terminal transmembrane and cytoplasmic domains of MLH1/ITGA9 interact with other cell proteins to exert their complex effect on the biology of the affected cell. Mismatch repair is abolished in tumor cells that are devoid of *MLH1* and carry at least one *MLH1/ITGA9* fusion copy, potentially leading to global genome instability^20^.

The *ITGA4* gene product is a component of two integrins, α4β1 and α4β7, and has been studied in detail to date. Yet, there is still no consensus as to the role integrin α4 plays in tumorigenesis. Some studies demonstrate that integrin α4 acts as a tumor suppressor, while others show that its expression correlate with the extent of cell malignant transformation and metastasis^22^. Abnormal *ITGA4* methylation has been observed in colorectal cancer^23,24^, cholangiocarcinoma^13^, gastric and bladder tumors^11^. Integrin α4β1 may promote melanoma cell adhesion to the vascular endothelium. Higher-level *ITGA4* expression may suppress the detachment and invasion of cancer cells and, on the other hand, may promote their dissemination by increasing their interaction with surface ligands of endothelial cells^25^.

Like integrins, dystroglycan also acts as a cell surface receptor. Dystroglycan consists of two subunits, α and β, which are synthesized as a propeptide from one mRNA; the propeptide is then cleaved proteolytically into two noncovalently associated subunits. Dystroglycan interacts with various extracellular matrix components, such as laminins, perlican, and agrin. In muscle cells, dystroglycan ensures physical contacts between the cytoskeleton and the basement membrane. Dystroglycan is also expressed in other cells and plays a role in epithelial morphogenesis, cell adhesion, and signal transduction^2^.

Studies of dystroglycan in breast tissue have shown its importance for maintaining the cell polarity, tissue architectonics, and β-casein secretion^26^. Diminished function of α-dystroglycan has been associated with a more aggressive tumor phenotype in BC cell lines^27^.

Nidogens are another family of essential components of the extracellular matrix. The family includes only two members, nidogens 1 and 2. Structurally, nidogens are 150-kDa sulfated glycorpoteins that consist of three globular domains. Nidogen surface presents binding sites for extracellular matrix proteins, such as laminins, collagens, and integrins. Nidogen-1, which is also known as entactin-1, interacts with integrins, perlican, and fibulin and can form a triple complex with collagen IV and laminin 111. The latter interaction underlies the formation of all basement membranes. Mesenchymal and myoepithelial cells express nidogen-1 in breast tissues. Nidogen-1 is thought to modulate the signals transmitted by laminin 111, in particular, to regulate β-casein expression^28^. Nidogen-2 interacts with the same matrix molecules as nidogen-1 with the exception of fibulin. In addition, nidogen-2 binds with endostatin and tropoelastin. Inactivation of nidogen-2 has led to a higher frequency of melanoma metastasis to the lung in experiments with mice^29^.

Nidogens play a role in epithelial morphogenesis by regulating cell adhesion and maintaining the basement membrane integrity and indirectly affect signal transduction^30^. Although nidogens are involved in these processes, little is still known today on their role in tumorigenesis. Negrini et al.^31^ have observed that the promoter regions of *NID1* and *NID2* are abnormally methylated in 67 and 29% of rectal cancer cases, respectively. Methylation of the two regions has additionally been detected in more than 90% of cases in gastric cancer. Abnormal methylation of the promoter regions in *NID1* and *NID2* is accompanied by lack of their expression in the MCF7, MDA-MB-231, and BT-20 BC cell lines. A demethylating agent has been shown to restore their expression in MDA-MB-231 cells^31^. It has recently been reported that endothelial cell-derived nidogen-1 inhibits migration of SK-BR-3 breast cancer cells^32^.

Methylation of the promoter region is one of the mechanisms that regulate gene expression. In this work, we have assessed methylation status of the promoter regions of the integrin, dystroglycan, and nidogen genes, and have evaluated the association of their abnormal hypermethylation with expression and epigenetic subtypes of breast cancer.

## Results

### DNA methylation at ITGA1, ITGA2, ITGA3, ITGA4, ITGA6, ITGA7, ITGA9, ITGB1, NID1, NID2, and DAG1 gene promoters in normal and malignant breast tissues and cell lines

Data for the integrin, nidogen, and dystroglycan genes 5′-cytosine-phosphate-guanine-3′ (CpG) islands assessed by methylation-sensitive restriction enzyme digestion PCR (MSRE-PCR) are summarized in Table 1. Based on the results of MSRE-PCR, the genes were classified into two categories: genes with CpG islands nonmethylated in normal breast tissues but prone to abnormal hypermethylation in BC (*ITGA1, ITGA4, ITGA7, ITGA9, NID1*, and *NID2*), and genes with CpG islands found to be nonmethylated in all breast tissues, no matter normal or cancerous (*ITGA2, ITGA3, ITGA6, ITGB1*, and *DAG1*).

**Table 1:** Methylation status of integrin, nidogen, and dystroglycan CpG islands assessed by MSRE-PCR in breast tissues and cell lines.

Gene	Methylated in breast cancer	Methylated in tissues adjacent to tumor	Methylated in normal autopsy breast tissues	Presence (+) or absence (−) of methylation in breast cancer cell lines				
				ZR751	MCF7	T47D	BT474	HS578T
*ITGA1*	13% (19/146)	1% (1/146)	0% (0/6)	−	−	−	−	−
*ITGA2*	0% (0/156)	0% (0/156)	0% (0/6)	−	−	−	−	−
*ITGA3*	0% (0/156)	0% (0/156)	0% (0/6)	−	−	−	−	−
*ITGA4*	31% (48/156)	3% (4/156)	0% (0/6)	+	+	+	+	−
*ITGA6*	0% (0/156)	0% (0/156)	0% (0/6)	−	−	−	−	−
*ITGA7*	4% (6/156)	1% (1/156)	0% (0/6)	−	−	−	+	−
*ITGA9*	39% (61/156)	2% (3/156)	0% (0/6)	+	+	+	+	−
*ITGB1*	0% (0/156)	0% (0/156)	0% (0/6)	−	−	−	−	−
*DAG1*	0% (0/156)	0% (0/156)	0% (0/6)	−	−	−	−	−
*NID1*	38% (59/154)	2% (3/156)	0% (0/6)	+	+	+	−	+
*NID2*	41% (63/153)	2% (3/156)	0% (0/6)	−	+	+	+	−

Breast cancer cell lines were analyzed alongside with the clinical samples in order to validate the results of our MSRE-PCR assays, and to provide reference information that can be reevaluated by other researchers. By now, the ENCODE project^33^ collection of data on DNA methylation obtained by reduced representation bisulfite sequencing (RRBS) contains information regarding two of the cell lines assessed in our study, MCF7 and T47D. For these, our MSRE-PCR results demonstrated in Table 1 recapitulate the ENCODE RRBS data for the *ITGA1, ITGA4, ITGA9, NID1* and *NID2* promoters approving the validity of our approach. *ITGA7* gene promoter is not covered by the ENCODE RRBS.

Nonmethylated status of the promoter CpG islands of the *ITGA1, ITGA2, ITGA3, ITGA4, ITGA6, ITGA9, ITGB1, NID1, NID2* and *DAG1* genes determined by MSRE-PCR in normal breast tissues in our study is in line with the ENCODE RRBS results for the normal breast tissue sample (BC_Breast_02-03015; breast, donor 02-03015, age 21, Caucasian, DNA extract). For *ITGA7* gene promoter, RRBS information is not available in the ENCODE.

Nonmethylated status of the promoter CpG islands of the *ITGA3, ITGA4, ITGA6, ITGA9, ITGB1, NID1, NID2* and *DAG1* genes in normal breast tissues is also supported by XmaI-RRBS previously performed by us on the same six autopsy samples^34^. *ITGA1*, *ITGA2* and *ITGA7* gene promoters are not covered with XmaI-RRBS data.

DNA methylated status of the regions assessed by MSRE-PCR was confirmed by a validating method of bisulfite sequencing by Sanger. Bisulfite Sanger sequencing was performed for the promoter of *ITGA7*, which evaded from ENCODE RRBS and XmaI-RRBS, to confirm its nonmethylated status in normal and tumor samples; and for one of the promoters (*NID2*) that demonstrated differential methylation by MSRE-PCR, to validate nonmethylated status in normal samples and abnormal methylation in cancer. *ITGA7* and *NID2* promoter regions were bisulfite sequenced in one normal sample and four tumor samples and the results confirmed MSRE-PCR findings in all cases.

### DNA methylation within a DAG1 gene intron

**Figure 2:**
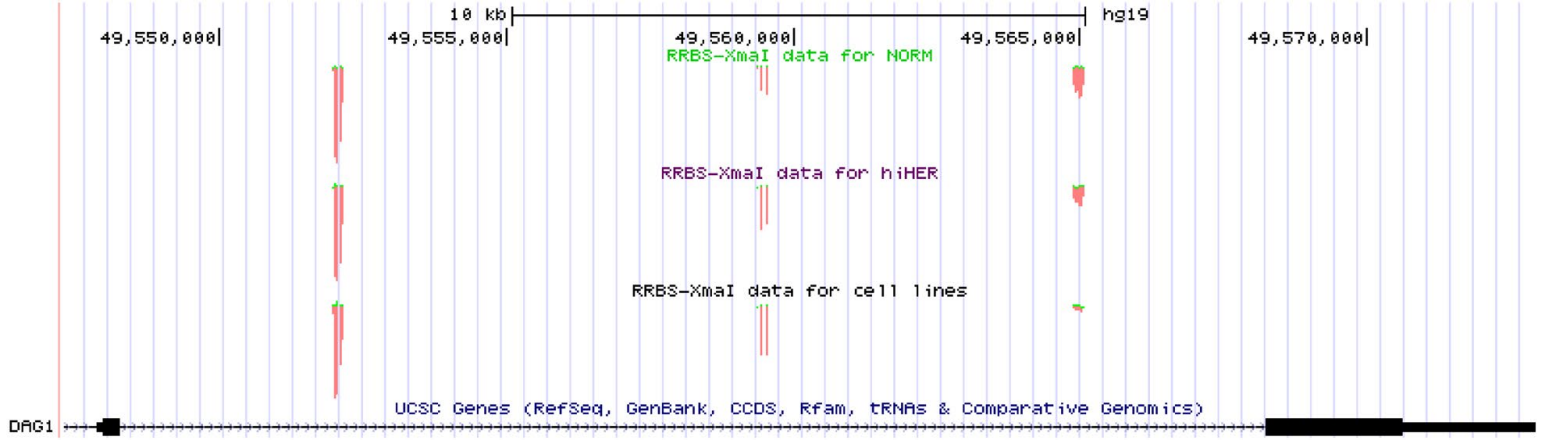
DNA methylation within a *DAG1* gene intron separating the two coding *DAG1* exons, assessed by XmaI-RRBS. The results are presented as a screenshot of our UCSC Genome Browser custom track that summarizes CpG methylation levels for all the samples that have been studied: normal breast tissues, upper track; BC tissues, middle track, BC cell lines, lower track. Prevalence of methylated CpGs in all three groups of samples is obvious from the prevalence of a fraction marked red. Nonmethylated fraction is marked green, and it is negligible. The two coding exons of the *DAG1* gene are schematically shown at the figure bottom.

Alongside with the gene promoters’ methylation information, our XmaI-RRBS results^34^ provide information on the methylation status of CpG-rich fragments of an intron separating the two coding exons of *DAG1*, one of the genes of interest in this study. The results presented on Fig. 2 demonstrate overwhelming prevalence of methylated alleles in BC samples and BC cell lines, as well as in normal breast tissues, indicating no or negligible evolutionary advantage of this area hypomethylation for breast cancer cells. Of note, this region is not covered in the available ENCODE project RRBS dataset^33^.

### Associations of hypermethylation of the ITGA1, ITGA4, ITGA9, NID1, and NID2 gene promoters and characteristics of breast tumors

Further analysis was focused on the *ITGA1, ITGA4, ITGA9, NID1*, and *NID2* genes in which we have identified CpGs nonmethylated in normal breast tissues but prone to abnormal hypermethylation in BC, with hypermethylation found in no less than 5% of samples from our collection.

**Figure 3:**
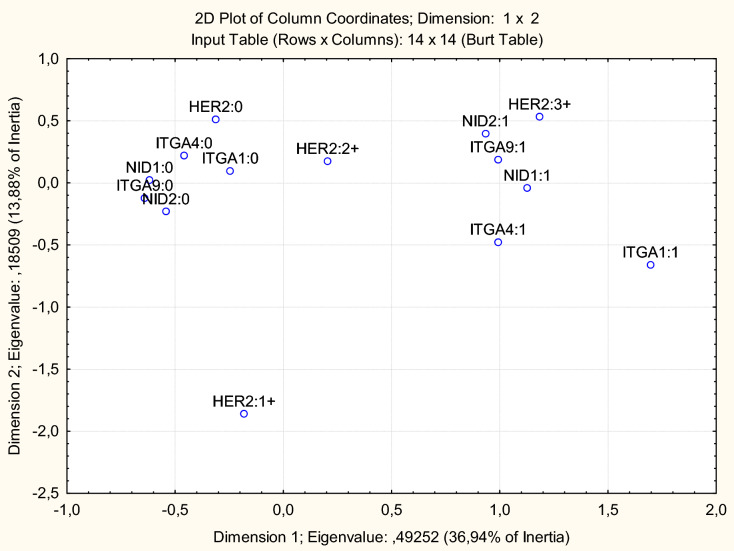
High HER2 expression (IHC score 3+) is associated with the methylated status of the *ITGA1, ITGA4, ITGA9, NID1*, and *NID2* genes in breast cancer samples by the results of multiple correspondence analysis. The nonmethylated gene status is designated as “0” at the gene symbol; methylated as “1” at the gene symbol. Multiple correspondence analysis allows investigating the structure of objects and variables that characterize them, initially included in the multi-input table, where objects (samples) are located in rows, and grouping variables (N) in columns, which are object coordinates in the N-dimensional space. The goal of the multiple correspondence analysis is to represent the contents of the table transformed into a table of relative frequencies of grouping variables as a projection on a 2-dimensional space. Dimension 1 explains 36.94% inertia, that is, for the data in question, the values of the relative frequencies that are reconstructed from one dimension contribute to the χ^2^ statistics (and, therefore, inertia) for 36.94% of the original. Both dimensions explain 50.82% of the χ^2^ value. Groups of samples that have signs of grouping variables located in the same fields are considered the closest. For example, on this plot, patients with a methylated status of the *ITGA9* and *NID2* genes and a high level of HER2 expression in breast cancer samples form a pronounced common cluster. A somewhat less pronounced commonality is demonstrated by the samples with methylated status of the *ITGA1*, *ITGA4*, and *NID1* genes.

A multiple correspondence analysis was carried out in order to suggest clinical and genetic associations between the methylation status of *ITGA1, ITGA4, ITGA9, NID1*, and *NID2* and clinical/morphological characteristics of the tumors, including the disease stage, tumor grade, tumor type, as well as estrogen receptor, progesterone receptor, and HER2 expression assessed by immunohistochemistry (IHC). By multiple correspondence analysis, potential association with abnormal methylation of *ITGA1, ITGA4, ITGA9, NID1*, and *NID2* was observed only for the HER2 expression level (Fig. 3).

**Figure 4:**
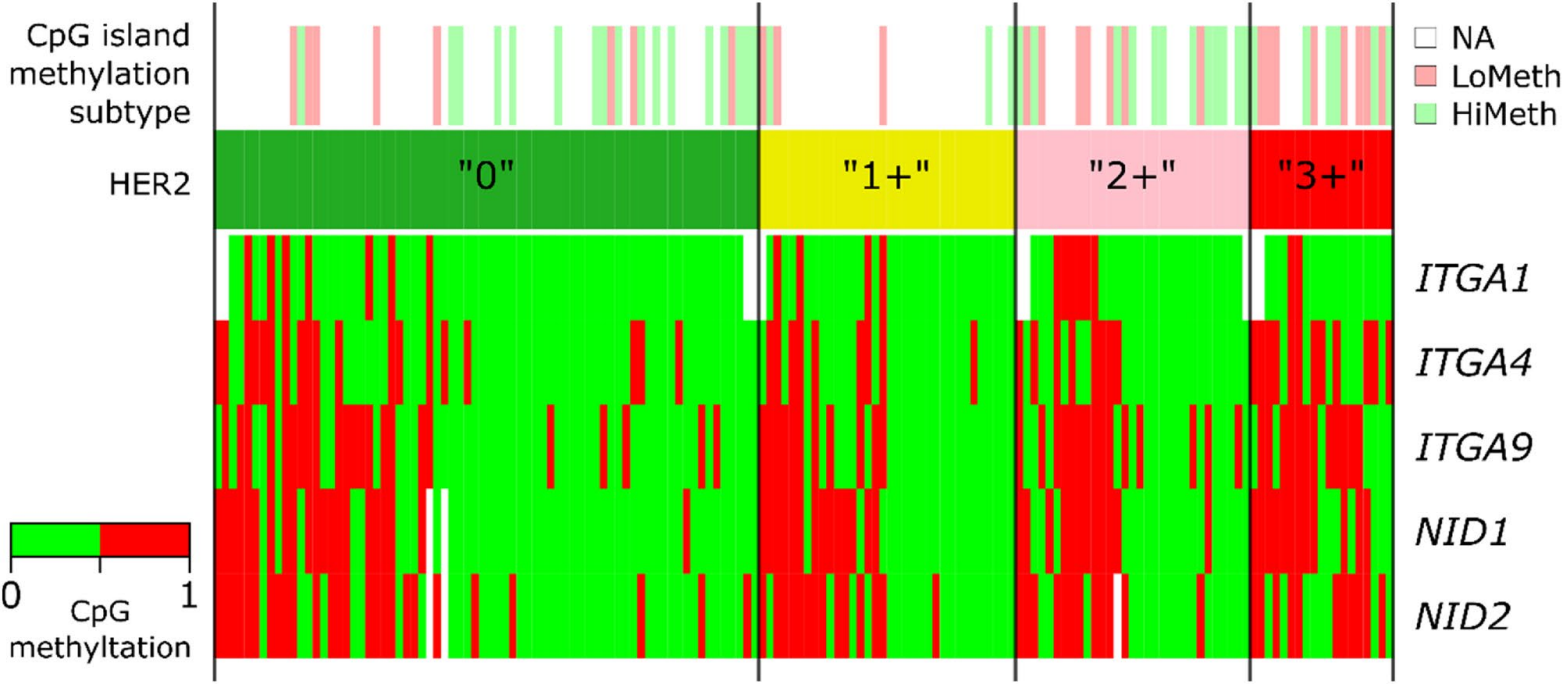
Promoter CpG methylation heatplot for the *ITGA1*, *ITGA4*, *ITGA9*, *NID1*, and *NID2* genes in the groups of breast cancer samples with different levels of HER2 expression designated as “0”, “1+”, “2+” and “3+” according to the IHC scores. LoMeth and HiMeth (CpG island methylation subtype track) are the moderately methylated and hypermethylated BC subtypes previously assigned to a subset of samples from the same tissue collection on the basis of a genome-wide DNA methylotyping^34^. NA, genome-wide DNA methylotyping data were not generated for the sample. The heatmap demonstrates methylated (red) or nonmethylated (green) statuses of the assessed promoter CpG dinucleotides. Blank fields stand for DNA methylation MSRE-PCR data missing for technical reasons.

Representations of BC samples with hypermethylated promoters in groups with different levels of HER2 expression are shown on Fig. 4. The association between the methylated gene status and high HER2 expression was confirmed by the χ^2^ and Fisher’s exact tests for *ITGA4, ITGA9*, and *NID1* (Table 2).

**Table 2:** Abnormal hypermethylation of CpG dinucleotides in the promoter regions of integrin and nidogen genes in breast cancer samples in relation to HER2 status.

Gene	HER2 expression, IHC score				Significant differences
	“0”	“1+”	“2+”	“3+”	
*ITGA1*	10% (7/68)	12% (4/33)	21% (6/28)	12% (2/17)	None
*ITGA4*	26% (19/72)	26% (9/34)	26% (8/31)	63% (12/19)	“0” vs “3+”, *p* = 0.0054
	26% (36/137)			63% (12/19)	“0, 1+, 2+” vs “3+”, *p* = 0.0025
*ITGA9*	32% (23/72)	32% (11/34)	48% (15/31)	63% (12/19)	None
	32% (34/106)		48% (15/31)	63% (12/19)	“0, 1+” vs “3+”, *p* = 0.0199
*NID1*	30% (21/70)	41% (14/34)	39% (12/31)	63% (12/19)	“0” vs “3+”, *p* = 0.017
*NID2*	37% (26/70)	41% (14/34)	40% (12/30)	58% (11/19)	None

We have also identified positive association of the CpG island hypermethylated status of the *ITGA1, ITGA9*, *NID1* and *NID2* genes in tumors with their attribution to the hypermethylated epigenomic BC subtype determined for a subset of samples from the same collection by genome-wide bisulfite sequencing of the CpG islands by XmaI-RRBS. Interestingly, we found no such association for the *ITGA4* gene (Table 3).

**Table 3:** Abnormal hypermethylation of CpG dinucleotides in the promoter regions of integrin and nidogen genes in breast cancer samples in relation to genome-wide tumor methylotype.

Gene	Genome-wide tumor methylotype		Significant differences
	Moderately methylated at CpG islands	Hypermethylated at CpG islands	Fisher’s exact test
*ITGA1*	0% (0/33)	22% (5/23)	*p* = 0.01
*ITGA4*	26% (10/38)	42% (11/26)	None
*ITGA9*	26% (10/38)	62% (16/26)	*p* = 0.01
*NID1*	16% (6/38)	65% (17/26)	*p* = 0.0002
*NID2*	19% (7/37)	69% (18/26)	*p* = 0.0002

**Figure 5:**
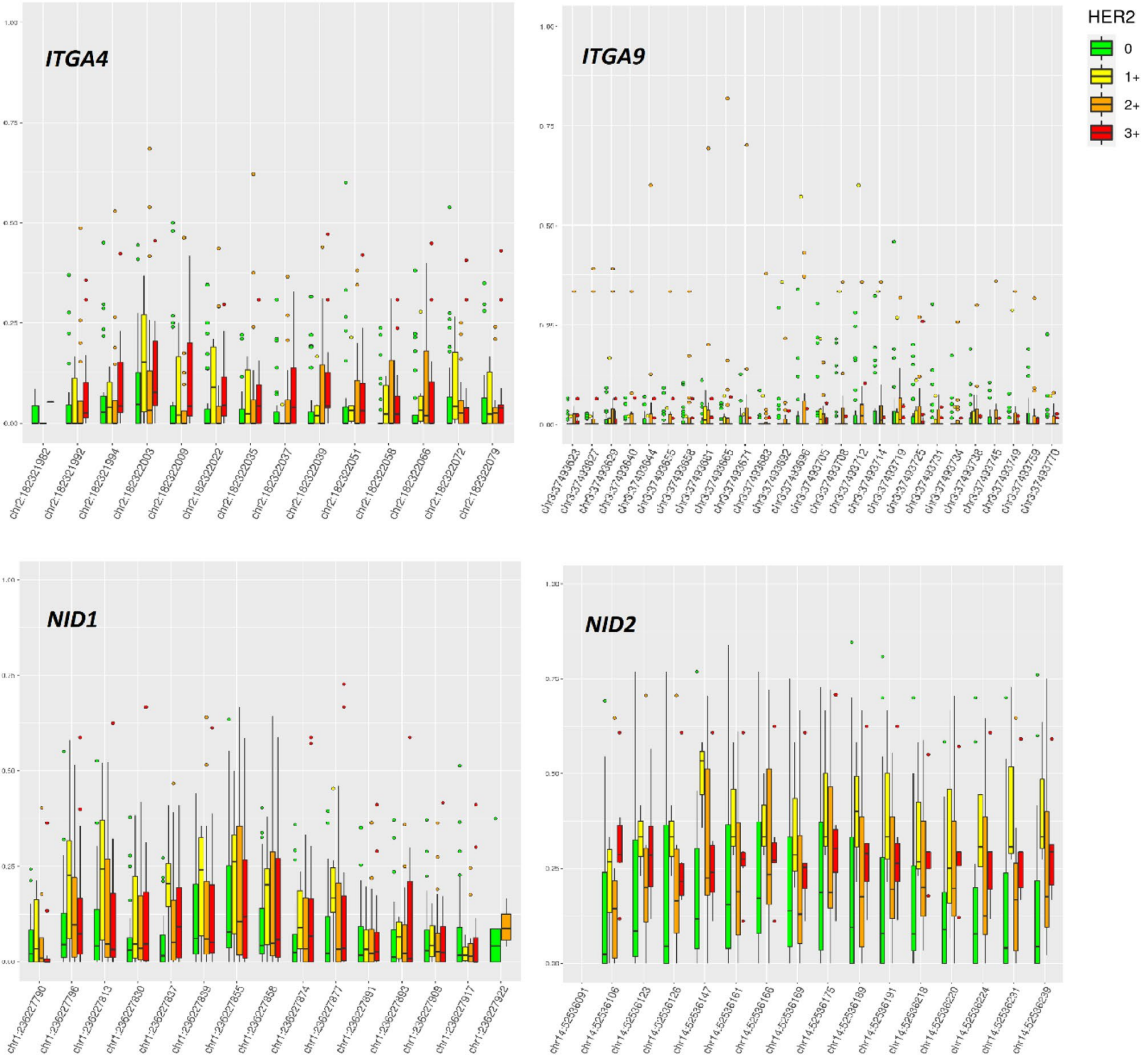
Relative fractions of methylated and nonmethylated alleles, measured in breast cancer samples by XmaI-RRBS, in respect to HER2 expression. Beta values ranging from 0.00 for totally nonmethylated to 1.00 for totally methylated (along the Y-axis) are the estimate of methylation level, and is the fraction of methylated CpG dinucleotides in all CpG dinucleotides assessed in genomic (GRCh37/hg19) positions indicated along the X-axis.

For the *ITGA4*, *ITGA9*, *NID1* and *NID2* genes for which XmaI-RRBS data are available, we have calculated the relative fractions of methylated and nonmethylated alleles in tumor samples, in respect to HER2 expression in tumors. The results are shown on Fig. 5.

## Discussion

We have previously examined DNA methylation at the CpG islands of all laminin subunit genes and have shown that the promoter regions of *LAMA1, LAMA2*, and *LAMB1* are affected by abnormal methylation in BC with the frequencies of 29%, 26% and 16% respectively^5^. The present study focuses on the genes for eleven proteins that are capable of interacting with the *LAMA1, LAMA2*, and *LAMB1* products.

We have studied the methylation status of eight integrin gene promoters in normal and BC cells, and observed abnormal hypermethylation in BC for *ITGA1, ITGA4, ITGA7*, and *ITGA9*.

*ITGA1* gene promoter was found to be abnormally hypermethylated in 13% (19/146) samples from our BC tissue collection. *ITGA1* is normally expressed in multiple tissues including breast. It was recently shown that *ITGA1* is a differentially expressed-aberrantly methylated gene in breast cancer, providing a possible independent functional role of its hypermethylation in BC^35^. In our study, *ITGA1* hypermethylation was not associated with HER2 tumor status, providing further evidence of its independent role in BC tumorigenesis.

**Figure 6:**
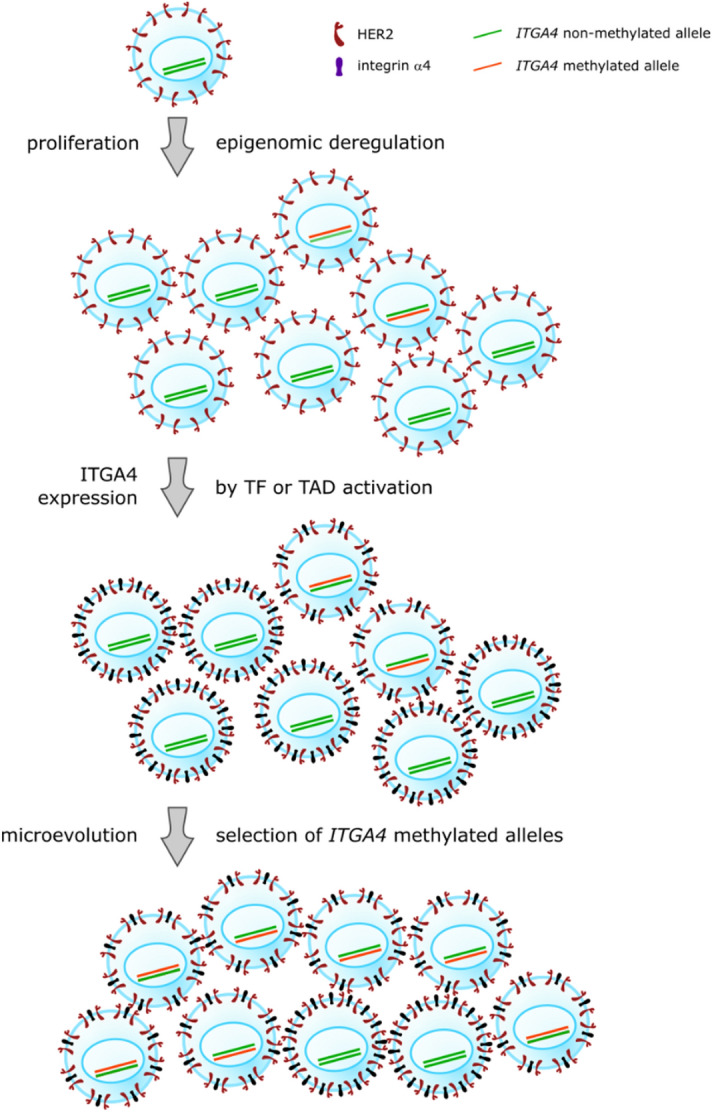
A hypothetical mechanism for the tumor microevolution advantage of the methylated *ITGA4* gene promoter in HER2 positive breast tumors. The hypothesis is that co-expression of the HER2 and integrin α4 receptors might reduce microevolution fitness of cancer cells; “preventive prohibition” of the *ITGA4* gene expression by its promoter methylation provides advantage for the cells in cases when under any circumstances the mechanisms of *ITGA4* transcription in a tumor are activated (see detailed explanation in the text). TF, transcription factor; TAD, topologically associating domain.

**Figure 7:**
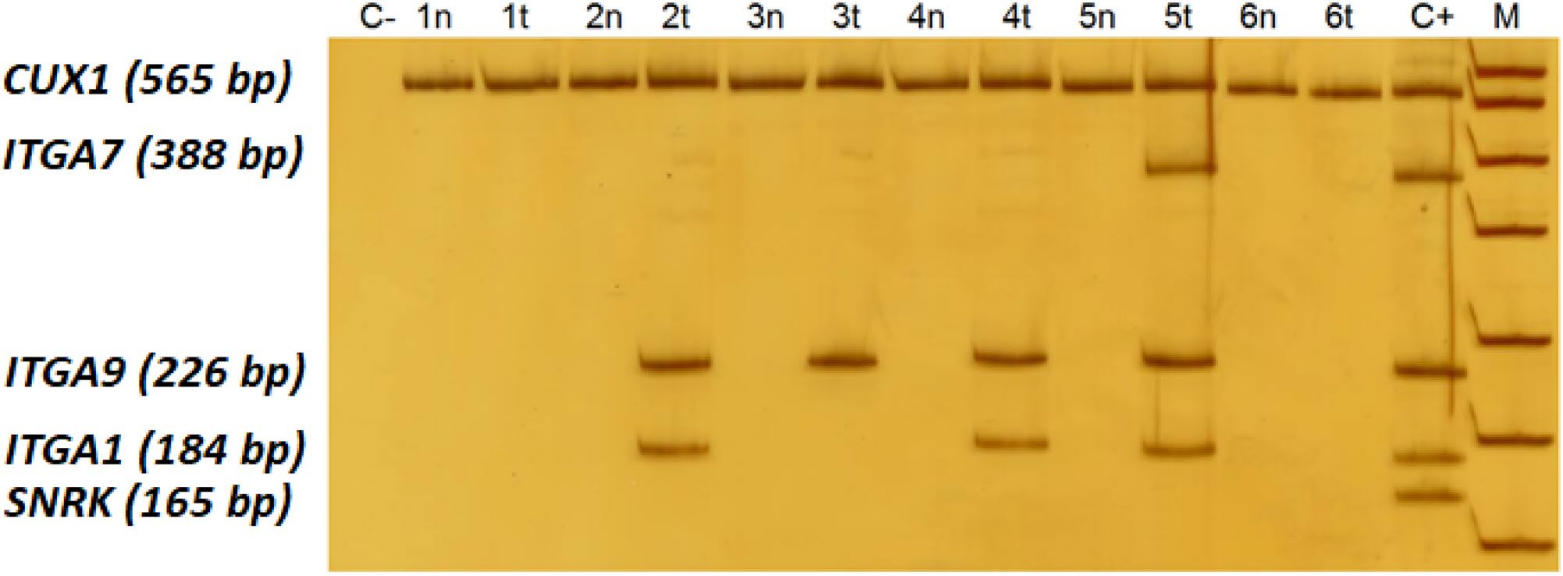
Simultaneous analysis of the fragments of the *ITGA1*, *ITGA7* and *ITGA9* genes promoters by MSRE-PCR. C-, negative MSRE-PCR control; 1–6, MSRE-PCR products obtained with breast cancer genomic DNA samples digested with HpaII, where “n” stands for the apparently normal morphologically intact tissue samples, and “t” stands for the matching tumor tissue samples; C+, MSRE-PCR products obtained with an undigested human genomic DNA as a template (positive sample control); M, DNA ladder pUC19/HpaII. Positions of the PCR products corresponding to the *ITGA1*, *ITGA7* and *ITGA9* promoter CpG islands under analysis, as well as a to a positive PCR control (a constitutively methylated region of the *CUX1* gene), and to a DNA digestion control (a constitutively nonmethylated region of the *SNRK* gene) are specified on the left. Samples 1 and 6 demonstrate nonmethylated status at the CpG islands of all the three target genes in both tumor and adjacent tissues; sample 2 shows methylated *ITGA1* and *ITGA9* and nonmethylated *ITGA7* in tumor tissue; sample 3 demonstrates methylation of *ITGA9* in tumor; sample 4 demonstrates methylation of *ITGA1* and *ITGA9* in tumor tissue; sample 5 shows methylation of all the three target genes in tumor sample. MSRE-PCR does not provide information on the methylation status of individual CpGs contained within the restriction enzyme recognition sequence in an assessed locus. Thus, positive MSRE-PCR signal was interpreted as hypermethylation of the whole target locus, while negative MSRE-PCR signal, as its nonmethylated state. The original full-length gel used for this Figure is shown on Supplementary Fig. S1.

The *ITGA4* gene promoter showed a high abnormal methylation frequency in our BC tissue collection (48/156 samples, 31%). Abnormal hypermethylation of this gene has previously been detected in 41% of tumor samples and was associated with the HER2 positive immunohistochemistry status and poor differentiation of tumor cells^14^. In the present study we also demonstrate high frequency of abnormal DNA methylation at the *ITGA4* gene promoter CpG island, also associated with the HER2 positive immunohistochemistry status. Do et al.^14^ speculate that such results support a previous study that suggested integrin α and HER2 are involved in the same signaling pathway, referring to the publication describing a novel mechanism for integrin-mediated Ras activation in breast carcinoma cells^36^. Yet, it should be kept in mind that the mentioned mechanism was described for integrin α6, as part of the α6β4 molecule. Both α6 and β4 integrin genes, *ITGA6* and *ITGB4*, are well expressed in the normal mammary tissues, unlike *ITGA4*, whose expression in normal breast is negligible (the gene expression data were obtained from the GTEx Portal https://www.gtexportal.org on 11/11/20). With this fact alone one should be cautious when drawing direct conclusions regarding common mechanisms of action of integrins α4 and α6. Further, the mechanism of integrin α6β4 action implies its involvement in HER2 expression. Depletion of β4 by shRNA reduced HER2 protein level without affecting *ERBB2* (HER2 gene) mRNA level and reexpression of β4 increased HER2 protein level^36^. These results show that integrin α6β4 positively regulates HER2 through translational control, and we thus should anticipate high α6β4 in the HER2 positive breast tumors. In the case of *ITGA4* in breast cancer, we witness the opposite situation, where the fraction of HER2 positive tumors is significantly enriched with samples harboring *ITGA4* methylation. If *ITGA4* is silenced by DNA methylation, as it is suggested by the results of several studies^11,14,24^, then integrin α4 should be low in HER2 positive tumors. Indeed, the question to discuss in the context of *ITGA4* abnormal hypermethylation in BC, is what the reason may be for the gene negligibly expressed in the normal tissue to become hypermethylated in the tumor of the same organ. We have recently suggested based on the examples of other extracellular matrix related molecules^37^, that cancer-specific abnormal hypermethylation of the genes that are not expressed in the same normal tissue might be a “passenger” epigenetic event reflecting merely a CIMP (CpG island methylator phenotype) of a tumor. Yet, while this may be an explanation for other genes found abnormally hypermethylated in BC samples in our study, as far as their hypermethylation well correlates with the CIMP phenotype (Table 3), this is not the case for *ITGA4* which is significantly more frequently found in tumors with moderate genome-wide levels of CpG islands methylation. Still, *ITGA4* hypermethylation is strongly associated with HER2 hyperexpression, and the association is more pronounced than for the other genes (Table 2). In terms of tumor evolution, this finding might reflect advantage for the cells in which elevation of *ITGA4* expression was occasionally “preventively prohibited” by its promoter methylation. Like any other CpG island, that of the *ITGA4* gene may acquire abnormal methylation in the process of cancer progression given general deregulation of epigenetic processes in tumors. If under any circumstances the mechanisms of *ITGA4* transcription in a tumor are activated (the activating mechanisms may be, for example, ectopic expression of a transcription factor, or activation of an otherwise inactive topologically associating domain), it would be more effective in the cells with nonmethylated *ITGA4* promoter then in those with the hypermethylated one. Then cells with nonmethylated *ITGA4* alleles would produce integrin α4 protein the function of which in cancer is not yet well understood but, judging by depletion of nonmethylated *ITGA4* alleles from the HER2 positive tumors, simultaneous presence of both HER2 and integrin α4 receptors is not beneficial for tumor cells (Fig. 6). This may suggest importance of HER2 and integrin α4 signaling pathways interactions that are yet to be discovered. Important though is the fact that in the HER2 positive tumors we never find hypermethylation of more than 50% of *ITGA4* alleles (Figs. 5, 7) and BC samples with completely nonmethylated *ITGA4* also exist, which suggests that simultaneous expression of HER2 and integrin α4 is not lethal, but rather renders some evolutionary disadvantage for the tumor cells that acquire such expression pattern.

*ITGA7* promoter CpG island was abnormally hypermethylated in 4% (6/156) samples in our study. Although The Cancer Genome Atlas data assessed by TCGA Wanderer^38^ indicate that *ITGA7* methylation at the region that we assessed tend to downregulate its expression, the number of hypermethylated samples is too small in our collection to draw any conclusions on the associations with clinical and molecular characteristics of the tumors.

In the present study, the *ITGA9* gene showed a high (39%) frequency of abnormal methylation in our collection of BC samples. This supports a previous report where in breast tumors, *ITGA9* expression was downregulated or totally absent in 44% of cases and intact or upregulated in 45% of cases, and the frequency of *ITGA9* promoter methylation was 90% in the former group; while treatment with the demethylating agent 5-aza-dC restored integrin α9 expression in ITGA9-negative cells of the MCF7 line^18^.

## Conclusions

In this study, we have identified high frequencies of cancer specific abnormal hypermethylation of the parts of promoter regions of integrin *ITGA1, ITGA4, ITGA9,* and nidogen *NID1*, *NID2* genes in breast cancer. Although their abnormal hypermethylation is cancer specific and frequent in breast tumors, which makes them attractive candidates for diagnostic markers, in the plain of tumor biology we suggest that this hypermethylation might be mere a “passenger” epigenetic event reflecting tumor CpG island methylator phenotype (CIMP). For the only gene among listed, *ITGA4*, abnormal hypermethylation is not correlated with CIMP, but is strongly correlated with high levels of HER2 expression in breast tumors. This finding may suggest importance of HER2 and integrin α4 signaling pathways interactions that are yet to be discovered. Data on possible involvement of receptors known to be overexpressed in breast cancer in regulation of integrin genes expression are gradually accumulating. It has very recently been suggested that estrogen receptor α is involved in the regulation of *ITGA8* methylation in estrogen receptor-positive breast cancer^39^. Further elaboration of this subject promises new exciting insights in cancer epigenomics.

## Materials and methods

### Clinical material

We examined 156 BC samples, 156 matched samples of morphologically normal adjacent tissue, five BC cell lines (ZR711, HS578T, BT474, T47D, and MCF7), and six autopsy samples of normal breast tissues. Biological material was obtained from the Blokhin Russian Cancer Research Center, Gertsen Moscow Research Cancer Institute, Research Centre for Medical Genetics, Institute of Gene Biology, and Russian Scientific Center of Roentgenoradiology. Altogether, 323 samples have been analyzed in this study. All BC and matched samples were obtained at surgery of cancer cases none of which underwent neoadjuvant chemotherapy. All tissue samples were fresh-frozen. Fragments of tissues for DNA analysis were examined macro- and micromorphologically: expression of ER, PR, and HER2 receptors was evaluated by immunohistochemistry. Negative HER2 status was diagnosed in 72 samples (46%); HER2 “1+”, “2+” and “3+” positivity was documented for 34 (22%), 31 (20%), and 19 (12%) samples, respectively.

The approximate amount of tumor cells in each sample was estimated to exceed 80%. Fragments of about 10 mg were used for DNA extraction.

This study was conducted in accordance with the Declaration of Helsinki, and was approved by the Bioethics Committee of Research Centre for Medical Genetics. Written informed consent was obtained from each participant of this study.

Of the 156 BC samples, 79% (123/156) were identified as ductal BC; 8% (13/156), as lobular BC; and 6% (9/156), as mixed BC. Micropapillary carcinoma and mucinous, medullary, metaplastic, and low-differentiated BC were diagnosed in single cases.

The disease stage at diagnosis was identified as I in 12% (19/156), IIA in 40% (63/156), IIB in 33% (51/156), IIIA in 6% (10/156), and IIIB in 6% (9/156), IIIC in 1% (2/156), and IV in 1% (2/156) of the patients.

### DNA isolation and methylation-sensitive restriction enzyme digestion

Genomic DNA was isolated by standard phenol–chloroform extraction. The DNA digestion mixture for the methylation-sensitive restriction enzyme digestion contained 1.5 μg of genomic DNA, 10 units of HpaII restriction endonuclease, and 2 μL of a SEBufferY buffer (10×) (SibEnzyme, Russia). Deionized water was added to bring the final volume to 20 μL, and the mixture was incubated at 37 °C for 16 h.

### Methylation-sensitive restriction enzyme digestion PCR (MSRE-PCR) assays

Triplex MSRE-PCR assays were used for each locus under study, where one fragment was amplified from the target gene, another one served as a positive PCR control (a constitutively methylated region of the *CUX1* gene^5^), and a third one was used to check the completeness of DNA hydrolysis (a constitutively nonmethylated region of *SNRK*^5^). Target loci with distinctly different amplicon lengths may be combined in the single-tube multiplex reactions (Fig. 5). The nucleotide sequences of the primers are shown in Table 4. PCR reactions were performed as described earlier^5^. The MSRE-PCR products were resolved by electrophoresis in 8% polyacrylamide gel and stained with silver nitrate (Fig. 7).

**Table 4:** Primers used to assess the methylation status of the integrin, nidogen, and dystroglycan genes by methylation-sensitive restriction enzyme digestion PCR.

Gene	Primers	Amplicon co-ordinates, GRCh37/hg19 [distance to transcription start site (TSS)]	PCR product size, bp	Number of HpaII sites within MSRE-PCR fragment
*ITGA1*	F: ATAAGTGGCCCAGCCAGAG R: GCAGGAGATGCTCAGAGAAAA	chr5:52,084,099–52,084,282 (includes TSS)	184	2
*ITGA2*	F: CCCCTAGGCACTGTGGTTTA R: AGGCTTTCCTGCCCTGGAC	chr5:52,284,728–52,284,933 (222 upstream)	206	2
*ITGA3*	F: AGCAGGTGAACAGGTCCTCA R: TACCAGGAATCGGGTATCCA	chr17:48,133,728–48,133,926 (388 bp downstream)	199	3
*ITGA4*	F: CCCCATCAGGTCCGCTCTTG R: CCCCACTCCCGGTTTCTGCC	chr2:182,321,921–182,322,166 (301 bp downstream)	246	6
*ITGA6*	F: CGAAGGTGGCTGCGGTAG R: CAGCGAGAAGCCGAAGAG	chr2:173,292,417–173,292,660 (103 bp downstream)	244	5
*ITGA7*	F: AATGAGGAGGCCCACAGAG R: CCAACGAGACTTTGGAGACC	chr12:56,101,182–56,101,569 (117 bp downstream)	388	3
*ITGA9*	F: GTTCTTCGGCTACGCAGTTC R: ACGGCGTCCTCCCTTCTC	chr3:37,494,003–37,494,228 (190 bp downstream)	226	3
*ITGB1*	F: GGGTCTGAGCACAAGCTG R: CAGTCCACTTCCCCGTGTT	chr10:33,246,321–33,246,420 (402 bp downstream)	100	3
*NID1*	F: GTCCTCCAGCTCCAGGTCCC R: CATCCCCGCCTTCCTCTGTC	chr1:236,228,236–236,228,440 (41 bp downstream)	205	4
*NID2*	F:GGAAGAGCTCGTCTGGGTGCAG R: CGCGGAGATCCAGGTTCGAG	chr14:52,535,601–52,535,810 (136 bp downstream)	210	4
*DAG1*	F: GCTGGATTGGCTGCAACACT R: GCAGGTGTTCGCTCCTCCTG	chr3:49,507,618–49,507,843 (53 bp downstream)	226	3
*CUX1*	F: GCCCCCGAGGACGCCGCTACC R: AGGCGGTCCAGGGGTCCAGGC	chr7:101,892,050–101,892,614 3′-UTR	565	6
*SNRK*	F: GCTGGGTGCGGGGTTTCGGCG R: CGGAGGCTACTGAGGCGGCGG	chr3:43,328,087–43,328,251 (84 bp downstream)	165	3

### Bisulfite sequencing by sanger

The results of the analysis of promoter methylation of target genes obtained by MSRE-PCR were verified with bisulfite sequencing of corresponding fragments. For bisulfite conversion, genomic DNA was denatured in NaOH (at a final concentration of 0.3 M) at 65 °C for 15 min. DNA was modified using sodium bisulfite and hydroquinone taken at final concentrations 2 M and 0.5 M, respectively, for 15 h at 55 °C. Modified DNA was purified using Wizard DNA Cleanup system (Promega, USA) according to the manufacturer’s instructions. PCR reactions were performed as described earlier^5^. PCR products were sequenced with an ABI3100 genetic analyzer using terminating dideoxynucleotides according to the protocol for ABI Prism 3100 Genetic Analyzer (Thermo Fisher Scientific, Waltham, Massachusetts, USA). The nucleotide sequences of the primers are shown in Table 5.

**Table 5:** Primers used to assess methylation of the *ITGA7* and *NID2* promoter regions by bisulfite Sanger sequencing.

Gene	Primers	Amplicon co-ordinates, GRCh37/hg19 [distance to transcription start site (TSS)]	PCR product size, bp
*ITGA7*	F: AGTAGGGAGTTAAAAAGGTAGTAAAT R: CCTAAAAAAACCTAAAACTTAAAA	chr12:56,101,399–56,101,540 (103 bp downstream)	142
*NID2*	F: GTGGGGTTTTTTGGGGGT R: CAATAACCACCACATCTAATTCTC	chr14:52,535,906–52,536,087 (includes TSS)	182

### Validation of MSRE-PCR results by RRBS

**Figure 8:**
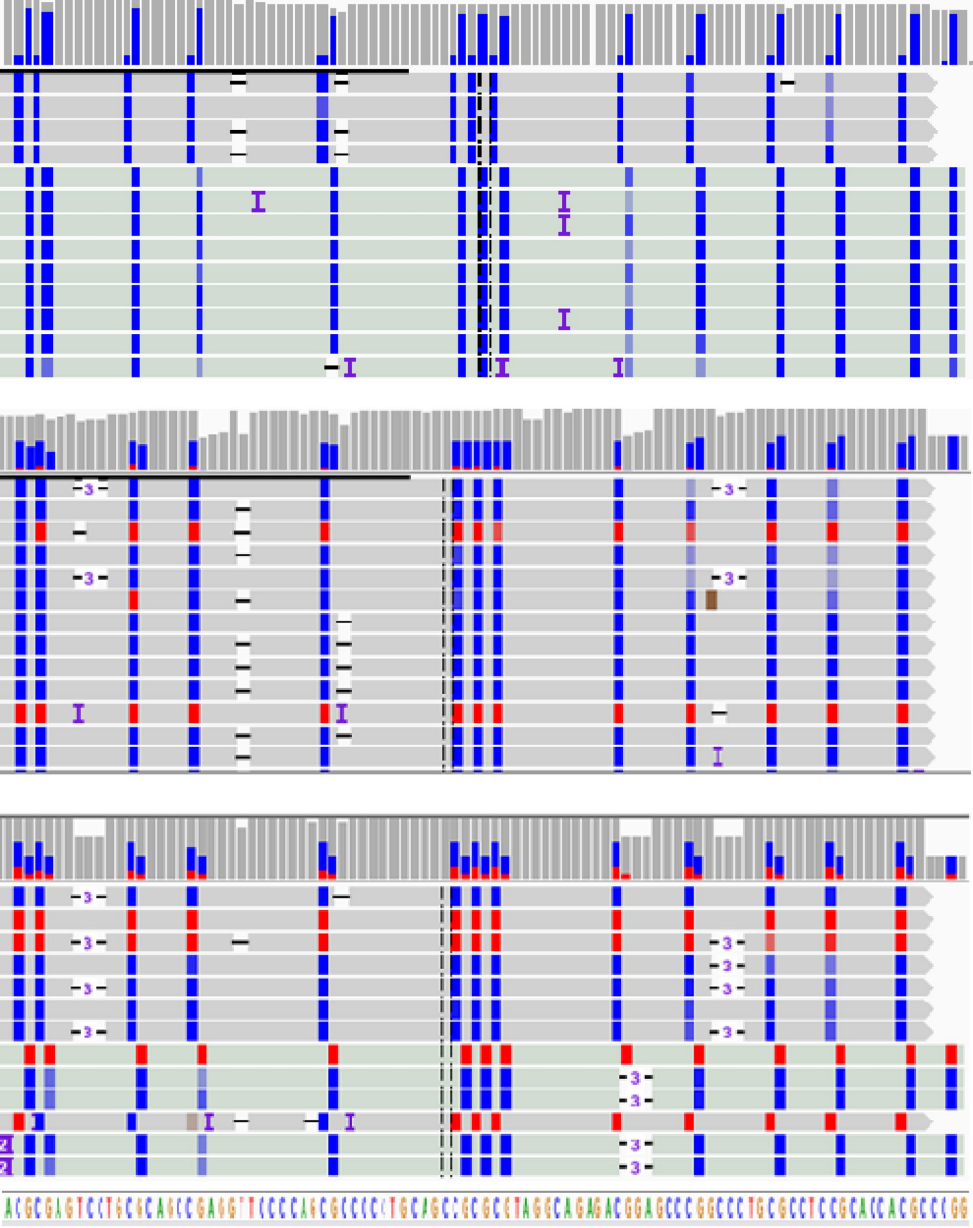
Methylation of a fragment of the *ITGA4* gene promoter assessed by reduced representation bisulfite sequencing (XmaI-RRBS). Each track represents a sequence (read) of a single molecule from the sample. A fragment of 96 base pairs is presented, sequenced in a normal breast tissue sample (upper panel) and in two different HER2 positive breast cancer samples (two lower panels). Red is for methylated cytosines and blue is for nonmethylated cytosines in the CpG context. Reference genome sequence is at the bottom. Symbols “-” and “-3-” seen within the reads reflect spurious single nucleotide and trinucleotide deletions, correspondingly, that arise as technical errors in detecting the lengths of homopolynucleotide tracts (poly-T and poly-A) arising as a result of bisulfite DNA conversion.

For the validation of MSRE-PCR results by RRBS, two RRBS datasets were used, one from the ENCODE project^33^, and another from our previous XmaI-RRBS study^34^ performed on a subset of 64 BC samples, five BC cell lines and six normal breast samples matching the samples from the collection described here. XmaI-RRBS was performed as described by us earlier^40^. A representative example of XmaI-RRBS results visualized with Integrative Genomic Viewer^41^ (IGV) is shown on Fig. 8.

### Statistical analysis

Statistical significance of associations between the methylated gene status and high HER2 expression was assessed by the χ^2^ and Fisher’s exact tests. Associations between the methylated gene status and genome-wide tumor methylotype were assessed by the Fisher’s exact test. Multiple correspondence analysis (Fig. 1) was carried out with Multiple Correspondence Analysis (MCA) software STATISTICA Version10. Distribution of methylation beta values in respect to HER2 expression in tumor samples was illustrated (Fig. 3) using ggplot2 package for R language. Influence of the methylation status of exact CpG dinucleotides on gene expression in BC was assessed using The Cancer Genome Atlas (TCGA) datasets using TCGA Wanderer^38^ at http://maplab.imppc.org/wanderer.

## Supplementary Information

Supplementary Information.
