# Supplementary material for: Abnormal promoter DNA hypermethylation of the integrin, nidogen, and dystroglycan genes in breast cancer
Source: Sci Rep. 2021 Jan 26;11:2264. doi: 10.1038/s41598-021-81851-y (PMC7838398; doi:10.1038/s41598-021-81851-y)
Supplement: Supplementary file 1 — Supplementary Information. [file 41598_2021_81851_MOESM1_ESM.docx]

Abnormal promoter DNA hypermethylation of the integrin, nidogen, and dystroglycan genes in breast cancer

Vladimir V. Strelnikov^1^*, Ekaterina B. Kuznetsova^1,3^, Alexander S. Tanas^1^, Viktoria V. Rudenko^2^, Alexey I. Kalinkin^1^, Elena V. Poddubskaya^4,5^, Tatiana V. Kekeeva^1^, Galina G. Chesnokova^1^, Ivan D. Trotsenko^6^, Sergey S. Larin^7,8^, Sergey I. Kutsev^1^, Dmitry V. Zaletaev^1,3^, Marina V. Nemtsova^1,3^ & Olga A. Simonova^2^

^1^ Epigenetics Laboratory, Research Centre for Medical Genetics, Moskvorechie St 1, 115522 Moscow, Russia;

^2^ Molecular Genetic Diagnostics Laboratory 2, Research Centre for Medical Genetics, Moskvorechie St 1, 115522 Moscow, Russia;

^3^ Medical Genetics Laboratory, I.M. Sechenov First Moscow State Medical University (Sechenov University), Trubetskaya St 8-2, 119991 Moscow, Russia

^4^ Clinic of Personalized Medicine, I.M. Sechenov First Moscow State Medical University (Sechenov University), Trubetskaya St 8-2, 119991 Moscow, Russia;

^5^ VitaMed LLC, Seslavinskaya St 10, 121309 Moscow, Russia

^6^ Institute of Medicine, Peoples' Friendship University of Russia (RUDN University), Miklukho-Maklaya St 6, 117198 Moscow, Russia;

^7^ Molecular Immunology Laboratory, Federal Scientific Clinical Centre of Pediatric Hematology Oncology Immunology Named after Dmitry Rogachev, Samory Mashela St 1, 117997 Moscow, Russia;

^8^ Gene Therapy Laboratory, Institute of Gene Biology, Vavilova St 34/5, 119334 Moscow, Russia

***** Corresponding author: vstrel@list.ru; Tel.: +7-926-235-2938

**Supplementary Figure S1:** Full-length gel (8% polyacrylamide gel stained with silver nitrate) used for Figure 7 in the article (see Figure 7 from the article below).

**
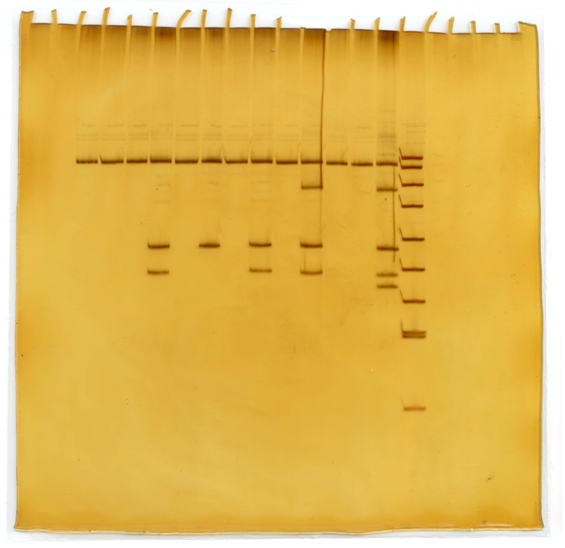
**


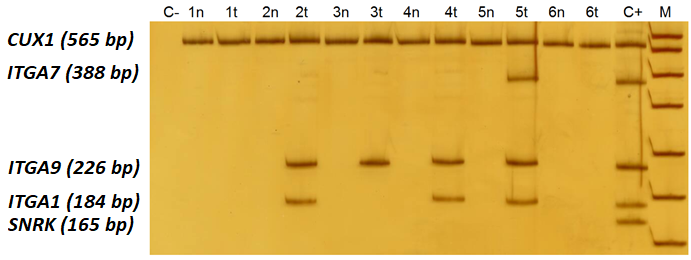


**Figure 7.** Simultaneous analysis of the fragments of the *ITGA1*, *ITGA7* and *ITGA9* genes promoters by MSRE-PCR. C-, negative MSRE-PCR control; 1–6, MSRE-PCR products obtained with breast cancer genomic DNA samples digested with HpaII, where “n” stands for the apparently normal morphologically intact tissue samples, and “t” stands for the matching tumor tissue samples; C+, MSRE-PCR products obtained with an undigested human genomic DNA as a template (positive sample control); M, DNA ladder pUC19/HpaII. Positions of the PCR products corresponding to the *ITGA1*, *ITGA7* and *ITGA9* promoter CpG islands under analysis, as well as a to a positive PCR control (a constitutively methylated region of the *CUX1* gene), and to a DNA digestion control (a constitutively nonmethylated region of the *SNRK* gene) are specified on the left. Samples 1 and 6 demonstrate nonmethylated status at the CpG islands of all the three target genes in both tumor and adjacent tissues; sample 2 shows methylated *ITGA1* and *ITGA9* and nonnmethylated *ITGA7* in tumor tissue; sample 3 demonstrates methylation of *ITGA9* in tumor; sample 4 demonstrates methylation of *ITGA1* and *ITGA9* in tumor tissue; sample 5 shows methylation of all the three target genes in tumor sample. MSRE-PCR does not provide information on the methylation status of individual CpGs contained within the restriction enzyme recognition sequence in an assessed locus. Thus, positive MSRE-PCR signal was interpreted as hypermethylation of the whole target locus, while negative MSRE-PCR signal, as its nonmethylated state.
